# Short- and mid-term outcomes of multisystem inflammatory syndrome in children: a longitudinal prospective single-center cohort study

**DOI:** 10.3389/fped.2023.1223266

**Published:** 2023-08-15

**Authors:** Ieva Roge, Anda Kivite-Urtane, Liene Smane, Anija Meiere, Lizete Klavina, Elza Barzdina, Jana Pavare

**Affiliations:** ^1^ Department of Pediatrics, Children’s Clinical University Hospital, Riga Stradins University, Riga, Latvia; ^2^ Department of Public Health and Epidemiology, Institute of Public Health, Riga Stradins University, Riga, Latvia; ^3^ Faculty of Medicine, Riga Stradins University, Riga, Latvia

**Keywords:** coronavirus disease 2019, multisystem inflammatory syndrome in children, multi-organ damage, pediatric, severe acute respiratory syndrome coronavirus 2

## Abstract

**Background:**

Multisystem inflammatory syndrome in children (MIS-c) emerged during the coronavirus disease 2019 pandemic and is associated with severe acute respiratory syndrome coronavirus 2 (SARS-CoV-2). Despite the extensively studied clinical manifestation of acute condition, the short- and long-term effects of MIS-c on children's health are unknown.

**Methods:**

This was a prospective longitudinal cohort study. Children aged <18 years who met the Centers for Disease Prevention and Control (CDC) diagnostic criteria and who were admitted to the Children's Clinical University Hospital of Latvia (CCUH) between July 1, 2020, and April 15, 2022, were enrolled in the study. An outpatient follow-up program was initiated in July 2020. All children were evaluated at 2 weeks, 2 months (1–3 months), and 6 months (5–7 months) after discharge. The face-to-face interviews comprised four domains as follows: symptom assessment, physical examination, laboratory testing, and cardiological investigation [including electrocardiogram (ECG) and echocardiography (echo)].

**Results:**

Overall, 21 patients with MIS-c were enrolled. The median age of the study group was 6 years. At the 2-week follow-up, almost half of the patients (*N* = 10, 47.6%) reported exercise intolerance with provoked tiredness. Laboratory tests showed a considerable increase in blood cell count, with a near doubling of leukocyte and neutrophil counts and a tripling of thrombocyte levels. However, a decline in the levels of inflammatory and organ-specific markers was observed. Cardiological investigation showed significant improvement with gradual resolution of the acute-phase pathological findings. Within 2 months, improvement in exercise capacity was observed with 5-fold and 2-fold reductions in physical intolerance (*N* = 2, 9.5%) and physical activity-induced fatigue (*N* = 5, 23.8%), respectively. Normalization of all blood cell lines was observed, and cardiological investigation showed no persistent changes. At the 6-month visit, further improvement in the children's exercise capacity was observed, and both laboratory and cardiological investigation showed no pathological changes.

**Conclusions:**

Most persistent symptoms were reported within the first 2 weeks after the acute phase, with decreased physical activity tolerance and activity-induced fatigue as the main features. A positive trend was observed at each follow-up visit as the spectrum of the children's complaints decreased. Furthermore, rapid normalization of laboratory markers and cardiac abnormalities was observed.

## Introduction

Multisystem inflammatory syndrome in children (MIS-c) is a potentially life-threatening condition that emerged during the global coronavirus disease 2019 (COVID-19) pandemic and is temporarily associated with severe acute respiratory syndrome coronavirus 2 (SARS-CoV-2). MIS-c develops 2–6 weeks after exposure to SARS-CoV-2 and is believed to be a post-infectious autoimmune condition characterized by hyperinflammation and multi-organ damage (1). The risk factors for MIS-c include young school age (6–11 years), male sex, chronic comorbidities, obesity, and ethnicity (Hispanic or non-Hispanic black children) (2–4).

The recent incidence rates varies from 30 to 45 per 100,000 children infected with SARS-CoV-2 (5, 6). However, new MIS-c cases have been reduced with each new SARS-CoV-2 variant emerging, suggesting that the new viral mutations, previously acquired immunity against SARS-CoV-2 and vaccination alter the inflammatory response in children, consequently reducing morbidity (7–9).

The hallmark of MIS-C includes systemic inflammation and multi-organ involvement, clinically presenting with fever, gastrointestinal, mucocutaneous, cardiovascular, respiratory, and neurocognitive symptoms, frequently phenotypically imitating complete/incomplete Kawasaki disease (KD) or toxic shock syndrome (TSS). Although MIS-c shares many clinical similarities to KD, considerable differences in epidemiological, laboratory and immunological factors exist (10–15). Approximately 70% of patients with MIS-c require admission to the pediatric intensive care unit (PICU) with inotropic and/or ventilatory support because of myocardial dysfunction, fluid-resistant shock, coronary artery aneurysms, or life-threatening arrhythmias (16, 17).

Although data on the epidemiological characteristics, clinical spectrum, pathophysiology, and treatment pathways of acute MIS-c have been studied extensively, information on the short- and long-term outcomes of MIS-c is still evolving. To date, some studies have followed up children after discharge from the hospital, generally revealing encouraging mid-term outcomes. However, fatigue, neurological sequelae, and emotional lability persisted in small subset of patients 6 months post-discharge (18, 19). The aim of this prospective cohort study was to this determine 6-month outcomes of MIS-c, by evaluating clinical, laboratory and cardiological data in real time.

## Materials and methods

This was a prospective longitudinal cohort study. Children aged <18 years, who fulfilled the Centers for Disease Prevention and Control (CDC) diagnostic criteria for MIS-c (20) and were admitted to the Children's Clinical University Hospital of Latvia (CCUH) between July 1, 2020, and April 15, 2022, were enrolled in the study. MIS-c cases were individually approved by a multidisciplinary team (pediatricians, infectologists, cardiologists, rheumatologists, and intensive care specialists) during hospitalization, according to the diagnostic criteria. Patients who did not meet the MIS-c criteria were excluded. Informed consent was obtained from all patients or their parents prior the participation in the study. This study protocol was reviewed and approved by the Ethics Committee of Riga Stradins University (approval no. 22-2/455/2021).

All children were admitted to the infectious disease ward or the PICU, depending on their general condition's severity. Treatment was immediately started after the diagnosis was established, according to the international guidelines for MIS-c treatment (21). Additionally, the medical team evaluated the treatment's effectiveness at different time points during hospitalization, taking into consideration the clinical changes in the child's general state of health, laboratory tests, and repeated cardiological investigation.

An outpatient follow-up program was established in July 2020 to evaluate the short- and long-term consequences of MIS-c. All patients were evaluated at 2 weeks, 2 months (1–3 months), and 6 months (5–7 months) after discharge from the hospital. Patients and their parents were interviewed during face-to-face visits using the symptom assessment form which was based on our previously developed questionnaire, evaluating long-COVID 19 symptoms in children (22). Additionally, a thorough physical examination, laboratory testing [including full blood count, C-reactive protein (CRP), interleukin-6 (Il-6), ferritin, D-dimers, fibrinogen, troponin I, creatine kinase -MB (CK-MB), albumin and lactate dehydrogenase (LDH)], as well as cardiological investigation (electrocardiogram and echocardiography) were performed at each visit. All visits were performed by a pediatrician and a cardiologist, thereby reducing the possibility of errors in data interpretation. Persistent symptoms were classified according to involved organ systems, including general sequelae, mucocutaneous, respiratory, cardiovascular, gastrointestinal, neurological, and cognitive complaints.

### Statistical analysis

Statistical analysis was performed using the Statistical Package for the Social Sciences (SPSS) version 26.0 (IBM SPSS Corp.). Statistical significance was set at *p* < 0.05. Descriptive statistics [medians and interquartile ranges (IQR)] and frequencies (expressed as percentages) were used for continuous and categorical variables, respectively. Friedman's test was used to detect the statistical significance of the trends in repeated measures of continuous variables over time (Wilcoxon test when compared in pairs), and Cochran's *Q* test was used for categorical variables (McNemar tests when compared in pairs).

## Results

### Demographic and acute hospitalization data

In total, 21 patients with confirmed MIS-c were included. The median age of the study group was 6 years (IQR, 5–10 years; range, 1–16 years). Regarding the age group distribution, most of the children were aged between 1 and 6 years (*N* = 11, 52.4%), with the remaining patients aged between 7 and 12 years (*N* = 9, 42.9%), and <13 years (*N* = 1, 4.8%). More than half of the patients (*N* = 11, 52.4%) were females. Two patients (9.5%) had known pre-existing comorbidities, including bronchial asthma and sensoneural hearing loss. Only fifteen (71.4%) patients had confirmed SARS-CoV-2 infection [positive polymerase chain reaction (PCR) test] before MIS-c development, whereas none had confirmed acute COVID-19 during admission. However, all patients (*N* = 21, 100%) had positive SARS-CoV-2 antibodies (anti-SARS-CoV-2 Spike IgG) in serum during admission, proving a recent infection. Furthermore, more than half of the children (*N* = 13, 61.9%) were admitted to the hospital from home, while the remaining (*N* = 8, 38.1%) were transferred from different regional hospitals. On average, the patients sought medical attention on the fourth day (IQR, 3–6 days) after the onset of acute symptoms.

All patients (*N* = 21, 100%) had fatigue and fever, with the median duration of fever being 8 days (IQR, 6.0–8.5 days). Additionally, all children had multi-organ involvement, including mucocutaneous, gastrointestinal, cardiovascular, and neurological symptoms, with polymorphous rash (*N* = 21, 100%), lethargy (*N* = 20, 95.2%), abdominal pain (*N* = 18, 85.7%), and tachycardia (*N* = 18, 85.7%) being the most frequent ones. During hospitalization, three patients (14.3%) complained of scalp dysesthesia without any evidence of underlying cutaneous disease.

In twelve patients (57.1%), electrocardiographic abnormalities, including ST-segment elevation (*N* = 10, 83.3%), heart rhythm abnormalities (*N* = 3, 25%), and transiently prolonged QTc intervals (*N* = 3, 25%) were detected. Additionally, more than half of all children (*N* = 11, 52.4%) had pathological echocardiography findings, with bilateral hydrothorax (*N* = 11, 52.4%), pericardial effusion (*N* = 9, 42.9%), left ventricular (LV) dysfunction (*N* = 9, 42.9%), and mitral/tricuspid regurgitation (*N* = 8, 38% each) being the most dominant findings. Three children (14.3%) had coronary artery dilatation (*z*-score: 2.0–2.5, according to the KD diagnostic guidelines) (23). More than half of the patients (*N* = 11, 52.4%) required admission to the PICU due to circulatory compromise, with a median of 2 days (IQR, 1.0–3.0) spent in the unit. The median length of hospital stay (LOS) was 12 days (IQR, 10–13 days). Table 1 outlines the demographic and clinical characteristics of the patients with MIS-c.

**Table 1:** Demographical data, acute symptom spectrum, and overall hospitalization data across the study group.

	MIS-c	
	*N*	%
Median (interquartile range) age, years	6.0 (5.0–10.0)	
Sex		
Male	10	47.6
Female	11	52.4
COVID-19 status		
PCR confirmed infection	15	71.4
Comorbidities	2	9.5
MIS-c symptoms in the acute phase	21	100.0
General symptoms (at least one symptom)	21	100.0
- Fever (>38.0°C)	21	100.0
- Median duration of fever	8.0 (6.0–8.5)	
- Fatigue	21	100.0
Mucocutaneous symptoms (at least one symptom)	21	100.0
- Mucositis	16	76.2
- Conjunctivitis	17	81.0
- Rash	21	100.0
Gastrointestinal symptoms (at least one symptom)		
- Nausea/vomiting	11	52.4
- Diarrhea	10	47.6
- Abdominal pain	18	85.7
Respiratory symptoms (at least one symptom)	21	100.0
- Cough	3	14.3
- Sore throat	2	9.5
- Shortness of breath	5	23.8
- Tachypnea	11	52.4
- Wheezing	0	0
- Hypoxia (SpO_2_ <92%)	4	19.0
Cardiovascular symptoms (at least one symptom)	21	100.0
- Peripheral edema (hands/feet)	11	52.4
- Tachycardia	18	85.7
- Hypotension	14	66.7
- Shock	2	9.5
Abnormal ECG	12	57.1
- ST-segment elevation	10	83.3
- Heart rhythm abnormalities	3	25.0
- Prolonged QTc interval	3	25.0
Pathological Echo findings	11	52.4
- Bilateral hydrothorax	11	52.4
- Pericardial effusion	9	42.9
- Left ventricular dysfunction	9	42.9
- Mitral regurgitation	8	38.0
- Tricuspid regurgitation	8	38.0
- Coronary artery dilatation	3	14.3
Neurological symptoms (at least one symptom)	21	100.0
- Headaches	15	71.4
- Lethargy	20	95.2
- Hyperesthesia	3	14.3
- Irritability	7	33.3
Hospitalization in ICU	11	52.4
Median days spent in PICU	2.0 (1.0–3.0)	
Median days spent in hospital	12.0 (10.0–13.0)	
Median days of symptoms at hospitalization	4.0 (3.0–6.0)	
Treatment		
- IVIG	20	95.2
- Glucocorticoids	19	90.5
- Glucocorticoids pulse therapy	5	23.8
- Anakinra	0	0
- Aspirin	21	100.0
- LMWH	20	95.2
- Inotropes	7	33.3
- Mechanical ventilation	0	0

MIS-c, multisystem inflammatory syndrome in children; PCR, polymerase chain reaction; COVID-19, coronavirus disease 2019; ICU, intensive care unit; PICU, pediatric intensive care unit; ECG, electrocardiography; Echo, echocardiography; LMWH, low-molecular-weight heparin; IVIG, intravenous immunoglobulin.

The bold values indicates have statistically significant results, where *p* value is below 0.05.

Almost all children (*N* = 20, 95.2%) received immunomodulatory therapy with high-dose intravenous immunoglobulin (IVIG). Eighteen children (85.7%) received IVIG in combination with low- to moderate-dose glucocorticoids (methylprednisolone 1–2 mg/kg/daily). Five children (23.8%) received high-dose glucocorticoid pulse therapy due to the refractory course of the disease. Nevertheless, none of the patients required intervention with biological medications. Low-molecular-weight heparin (LMWH) and aspirin were introduced in all patients according to the guidelines (19). Additionally, all children received antibacterial treatment because of a possible differential diagnosis of severe bacterial infection and TSS until all microbiological cultures returned negative results. Most used antibiotics were cefuroxime (*N* = 8, 38.1%), ceftriaxone (*N* = 8, 38.1%), cefotaxime (*N* = 3, 14.3%), and clindamycin (*N* = 2, 9.5%). Five children (23.8%) received combined antibacterial treatment with cephalosporins and clindamycin.

### Two weeks outcome

All children were examined 2 weeks after the MIS-c diagnosis was established. The patients were hemodynamically stable during the evaluation and showed significant clinical improvement after receiving immunomodulatory therapy. However, the most persistent complaint was decreased physical activity. Approximately half of the children (*N* = 10, 47.6%) reported exercise intolerance, which provoked tiredness after any exercise. Moreover, three children (14.3%) had difficulty walking for more than 15 min and climbing the stairs. Some of the patients also reported symptoms such as shortness of breath at rest (*N* = 5, 23.8%), fatigue (*N* = 4, 19%), irritability, and difficulty in concentrating (*N* = 2, 9.5% each). One patient (4.8%) reported persistent abnormal sensations on the scalp with accompanied headaches. However, no pathological clinical findings other than lymphadenopathy were observed during the physical examination. Table 2 highlights the persistent symptom spectrum across different time points. All symptoms, excluding cough, shortness of breath at rest, and irritability, were more likely to be associated with acute MIS-c phase than with short-term symptom persistence (*p* < 0.05). Additionally, decreased physical activity tolerance was a characteristic complaint at the 2-week follow-up rather than the 2- or 6-month follow-up visit (*p* < 0.05) (Table 3).

**Table 2:** Prevalence of reported symptom spectrum in acute MIS-c and different follow-up time-points.

Symptoms	Acute phase		2-week follow-up		2-months follow-up		6-months follow-up		*p*
	*N*	%	*N*	%	*N*	%	*N*	%	
General symptoms									
- Fatigue	21	100.0	4	19.0	0	0	2	9.5	**≤0** **.** **001**
- Elevated body temperature	21	100.0	0	0	2	9.5	0	0	**≤0** **.** **001**
Mucocutaneous symptoms									
- Rash	21	100.0	0	0	0	0	0	0	**≤0** **.** **001**
- Mucositis	16	76.2	0	0	0	0	0	0	**≤0** **.** **001**
- Peripheral edema (hands/feet)	11	52.4	0	0	0	0	0	0	**≤0** **.** **001**
- Lymphadenopathy	18	85.7	3	14.3	0	0	0	0	**≤0** **.** **001**
Gastrointestinal symptoms									
- Nausea/vomiting	11	52.4	0	0	1	4.8	0	0	**≤0** **.** **001**
- Diarrhea	10	47.6	0	0	0	0	0	0	**≤0** **.** **001**
- Weight changes	-	-	-	-	5	23.8	4	19.0	0.99
Respiratory symptoms									
- Cough	3	14.3	0	0	0	0	0	0	**0** **.** **03**
- Breathlessness at rest	5	23.8	5	23.8	0	0	0	0	**0** **.** **01**
- Wheezing	0	0	0	0	0	0	0	0	-
- Rhinorrhoea	1	4.8	1	4.8	0	0	0	0	0.57
- Tachypnea	11	52.4	0	0	0	0	0	0	**≤0** **.** **001**
Cardiovascular symptoms									
- Tachycardia	18	85.7	0	0	0	0	0	0	**≤0** **.** **001**
- Decreased physical activity tolerance	-	-	10	47.6	2	9.5	2	9.5	**0** **.** **002**
- Physical activity provoked tiredness	-	-	10	47.6	5	23.8	2	9.5	**0** **.** **02**
- Difficulties in walking for >15 min	-	-	3	14.3	1	4.8	1	4.8	0.37
- Difficulties in getting to the 3rd floor	-	-	3	14.3	1	4.8	2	9.5	0.55
Neurological symptoms									
- Headaches	15	71.4	1	4.8	1	4.8	1	4.8	**≤0** **.** **001**
- Hyperesthesia	3	14.3	1	4.8	1	4.8	1	4.8	0.39
Cognitive symptoms									
- Difficulties to concentrate	-	-	2	9.5	2	9.5	2	9.5	0.99
- Impaired memory	-	-	1	4.8	1	4.8	1	4.8	0.99
- Mood swings	-	-	1	4.8	1	4.8	1	4.8	0.99
- Irritability	7	33.3	2	9.5	1	4.8	1	4.8	**0** **.** **02**
- Depression/anxiety	-	-	0	0	1	4.8	0	0	0.37

MIS-c, multisystem inflammatory syndrome in children.

**Table 3:** Statistical significance of the comparisons of the symptom frequencies between different follow-up time points.

	Acute phase vs. 2 weeks	Acute phase vs. 2 months	Acute phase vs. 6 months	2 weeks vs. 2 months	2 weeks vs. 6 months	2 months vs. 6 months
General symptoms						
Fatigue	**<0** **.** **001**	**<0** **.** **001**	**<0** **.** **001**	0.13	0.69	0.50
Elevated body temperature	**<0** **.** **001**	**<0** **.** **001**	**<0** **.** **001**	0.50	-	0.50
Mucocutaneous symptoms						
Rash	**<0** **.** **001**	**<0** **.** **001**	**<0** **.** **001**	-	-	-
Mucositis	**<0** **.** **001**	**<0** **.** **001**	**<0** **.** **001**	-	-	-
Peripheral edema (hands/feet)	**0** **.** **001**	**0** **.** **001**	**0** **.** **001**	-	-	-
Lymphadenopathy	**<0** **.** **001**	**<0** **.** **001**	**<0** **.** **001**	0.25	0.25	-
Gastrointestinal symptoms						
Nausea/vomiting	**0** **.** **001**	**0** **.** **01**	**0** **.** **001**	0.99	-	0.99
Diarrhea	**0** **.** **002**	**0** **.** **002**	**0** **.** **002**	-	-	-
Respiratory symptoms						
Cough	0.25	0.25	0.25	-	-	-
Breathlessness at rest	0.99	0.06	0.06	0.06	0.06	-
Tachypnea	**0** **.** **01**	**0** **.** **01**	**0** **.** **01**	-	-	-
Cardiovascular symptoms						
Tachycardia	**<0** **.** **001**	**<0** **.** **001**	**<0** **.** **001**	-	-	-
Decreased physical activity tolerance	-	-	-	**0** **.** **01**	**0** **.** **01**	0.99
Physical activity provoked tiredness	-	-	-	0.23	**0** **.** **01**	0.45
Neurological symptoms						
Headaches	**<0** **.** **001**	**<0** **.** **001**	**<0** **.** **001**	0.99	0.99	0.99
Cognitive symptoms						
Irritability	0.13	0.07	0.03	0.99	0.99	0.99

The bold values indicates have statistically significant results, where *p* value is below 0.05.

Comparing the 2-week laboratory data to the acute MIS-c phase, a considerable increase in blood cell count was observed, with a near doubling of leukocyte [7.8 × 10^3^ (IQR, 6.1–14.1) vs. 14.9 × 10^3^ (12.8–19.4)] and neutrophil [5.6 × 10^3^ (IQR 4.7–11.0) vs. 9.4 × 10^3^ (IQR, 6.3–12.4)] counts, and tripling of thrombocyte levels [169 × 10^9^ (IQR, 127.5–232.0) vs. 625 × 10^9^ (IQR, 481.0–712.5)]. In contrast, considerable declines in the levels of inflammatory and organ-specific markers (CRP, Il-6, ferritin, troponin I, LDH, D-dimers, and fibrinogen) were observed (Table 4). As presented in Table 5, MIS-c was characterized by a notable increase in inflammatory and organ-specific markers (*p* < 0.05) when compared with the follow-up.

**Table 4:** Laboratory results at the baseline and at 2 weeks’, 2 months’, 6 months’ follow-up.

Parameters	Reference intervals of tested laboratory parameters in CCUH	In the acute phase (Median, IQR)	Follow-up at 2 weeks (Median, IQR)	Follow-up at 2 months (Median, IQR)	Follow-up at 6 months (Median, IQR)	*p*-value
		*N* = 21	*N* = 21	*N* = 21	*N* = 21	
Total WBC count (10^5^)	-	7.8 (6.1–14.1)	14.9 (12.8–19.4)	7.8 (6.3–10.3)	7.4 (6.7–8.7)	**≤0** **.** **001**
Total neutrophil count (10^5^)	-	5.6 (4.7–11.0)	9.4 (6.3–12.4)	3.4 (2.4–4.3)	3.1 (2.5–4.5)	**≤0** **.** **001**
Total thrombocyte count (10^5^)	-	169.0 (127.5–232.0)	625.0 (481.0–712.5)	369.0 (292.0–452.5)	338.0 (268.0–444.5)	**≤0** **.** **001**
CRP (mg/L)	0–2.8	138.0 (104.0–238.5)	2.1 (1.0–4.8)	0.4 (0.2–1.1)	1.5 (0.6–3.0)	**≤0** **.** **001**
Il-6 (pg/ml)	0–5.9	162.0 (88.4–254.0)	2.0 (2.0–2.0)	2.0 (2.0–2.0)	2.0 (2.0–2.0)	**≤0** **.** **001**
Ferritin (ng/ml)	150–450	434.5 (251.8–653.2)	265.0 (163.8–470.0)	27.1 (15.7–37.3)	33.7 (21.4–53.1)	**≤0** **.** **001**
LDH (U/L)	120–300	275.0 (244.0–342.0)	254.0 (220.3–272.0)	226.0 (183.8–263.8)	239.0 (190.0–255.5)	**0** **.** **01**
Troponin I (ng/ml)	0–12	30.0 (12.9–49.0)	3.2 (1.5–5.4)	1.4 (0.5–2.7)	0.2 (0.1–0.8)	**≤0** **.** **001**
CK-MB (ng/ml)	0–5.1	0.8 (0.3–1.0)	0.6 (0.2–0.8)	1.0 (0.8–1.7)	0.8 (0.7–2.0)	0.13
D-dimers (mg/L)	0–0.55	3.3 (2.2–5.8)	0.8 (0.4–1.3)	0.2 (0.2–0.2)	0.2 (0.2–0.3)	**≤0** **.** **001**
Fibrinogen (g/L)	1.7–4.2	4.4 (3.6–5.3)	1.7 (1.6–2.2)	2.5 (1.9–2.9)	2.0 (1.8–2.8)	**≤0** **.** **001**
Albumin (g/L)	32–45	30.7 (26.1–34.9)	37.3 (35.5–38.1)	46.5 (44.4–48.4)	46.0 (43.8–48.7)	**≤0** **.** **001**

CRP, C-reactive protein; LDH, lactate dehydrogenase; CCUH, Children's Clinical University Hospital of Latvia; IQR, interquartile range; CK-MB, creatine kinase-MB; WBC, white blood cell; IL-6, interleukin-6.

The bold values indicates have statistically significant results, where *p* value is below 0.05.

**Table 5:** Statistical significance of the comparisons of the laboratory finding frequencies between different follow-up time points.

	Acute phase vs. 2 weeks	Acute phase vs. 2 months	Acute phase vs. 6 months	2 weeks vs. 2 months	2 weeks vs. 6 months	2 months vs. 6 months
Total WBC count (10^5^)	**0** **.** **01**	0.41	0.16	**<0** **.** **001**	**<0** **.** **001**	0.55
Total neutrophil count (10^5^)	0.31	**<0** **.** **001**	**<0** **.** **001**	**<0** **.** **001**	**<0** **.** **001**	0.91
Total thrombocyte count (10^5^)	**<0** **.** **001**	**<0** **.** **001**	**<0** **.** **001**	**<0** **.** **001**	**<0** **.** **001**	0.10
CRP (mg/L)	**<0** **.** **001**	**<0** **.** **001**	**<0** **.** **001**	**0** **.** **01**	0.39	0.10
Il-6 (pg/ml)	**<0** **.** **001**	**0** **.** **001**	**<0** **.** **001**	0.32	0.11	0.18
Ferritin (ng/ml)	**0** **.** **002**	**<0** **.** **001**	**<0** **.** **001**	**<0** **.** **001**	**<0** **.** **001**	0.20
LDH (U/L)	**0** **.** **01**	**0** **.** **001**	**<0** **.** **001**	0.57	0.14	0.83
Troponin I (ng/ml)	**<0** **.** **001**	**<0** **.** **001**	**<0** **.** **001**	0.10	**0** **.** **01**	**0** **.** **03**
D-dimers (mg/L)	**<0** **.** **001**	**<0** **.** **001**	**<0** **.** **001**	**<0** **.** **001**	**<0** **.** **001**	0.14
Fibrinogen (g/L)	**0** **.** **002**	**0** **.** **001**	**<0** **.** **001**	**0** **.** **02**	0.27	0.08
Albumin (g/L)	**0** **.** **004**	**<0** **.** **001**	**<0** **.** **001**	**0** **.** **002**	**0** **.** **001**	0.49

CRP, C-reactive protein; LDH, lactate dehydrogenase; WBC, white blood cell; IL-6, interleukin-6.

The bold values indicates have statistically significant results, where *p* value is below 0.05.

All patients also underwent a cardiological investigation, and gradual improvement was observed. Repeated ECG resolutions of ST elevations and rhythm abnormalities were observed. However, only one patient (8.3%) had a persistently prolonged QTc interval, with a tendency to decrease. Additionally, the echo showed improved heart function with the disappearance of valvular insufficiency and fluid collections in the pleura and pericardium. All children with coronary artery involvement showed good improvement, with z-score normalization. The tapering doses of oral glucocorticoids and aspirin were continued until the next visit.

### Two months follow-up

All patients were repeatedly evaluated following the developed protocol at 2 months (1–3 months). During the follow-up visit, all children were generally well and had resumed their normal daily activities, including school and daycare attendance. After their discharge from the hospital, three children (14.3%) developed acute respiratory viral infections, and they were all treated at home. Approximately one-quarter of parents (*N* = 5, 23.8%) during the interview reported significantly increased appetite and weight gain of their child since discharge from the hospital. However, five children (23.8%) still had physical activity-induced tiredness, with only two (9.5%) reporting considerably decreased physical activity tolerance. Two children (9.5%) had periodically prolonged elevated body temperature (approximately 37.6°C) without any other complaints. One female patient (4.8%) still had persistent scalp dysesthesia and headaches. Like the 2-week follow-up, all symptoms excluding cough, shortness of breath at rest, and irritability, were more consistent with acute MIS-c rather than persistent complaints at the 2-month follow-up (*p* < 0.05) (Table 3).

Analysis of laboratory data showed the normalization of all blood cell lines. Specifically, all inflammatory and organ-specific markers were in the normal range, and the albumin level returned to normal [46.5 g/L (IQR, 44.4–48.4)]. Compared with the 2-week follow-up, a significant decrease was observed in ferritin level (265.0 ng/ml (IQR, 163.8–470.0) vs. 27.1 ng/ml (IQR, 15.7–37.3) with normal hemoglobin levels and no signs of anemia.

All children were consulted by a cardiologist during the follow-up, and ECG and echo were performed. However, no pathology was observed in any of the examinations, and aspirin therapy was discontinued in all children.

### Six months follow-up

All children were healthy at 6 months (5–7 months) without any major health-related issues. However, seven children (33.3%) had mild to moderate acute viral respiratory infection episodes since the last follow-up and were treated in an outpatient setting. Four children (19%) still showed progressive weight gain, whereas two (9.5%) had decreased physical activity tolerance. Reduction in physical activity-induced tiredness was observed in the 6-month follow-up, when compared with the 2-month follow-up (*N* = 5, 23.8% vs. *N* = 2, 9.5%) data. Two children (9.5%) reported fatigue which they did not report during previous visits. In contrast, patients who reported persistent fatigue at the 2-week visits returned to normal energy levels and denied any signs of persistent fatigue. Patients with scalp dysesthesia (*N* = 1, 4.8%) reported symptom persistence 6 months after acute MIS-c onset.

However, no pathological changes were observed in laboratory tests. Ferritin level was slightly higher [27.1 ng/ml (IQR, 15.7–37.3) vs. 33.7 ng/ml (IQR, 21.4–53.1)] in the 6-month follow-up compared with the 1–3 months visit. Moreover, the cardiological investigation showed no abnormal findings.

## Discussion

This longitudinal prospective cohort study described and analyzed the clinical and laboratory course of MIS-c from the onset of an acute condition up to 6 months after hospitalization in 21 children to elucidate possible post-inflammatory sequelae. Our study showed that most patients had short-term subjective complaints after acute MIS-c, with decreased physical activity tolerance and activity-provoked fatigue as the main features.

Several attempts have been made to identify the short- and long-term outcomes of MIS-c (18, 19, 24–26). Generally, a common conclusion can be deduced from these published studies that patients with MIS-c generally recover well, leaving no or few short- and long-term consequences, despite the severe clinical picture in the acute phase.

Compared with other studies, our cohort's demographic profile was partly like those reported in other studies, with similar male predominance but lower median patient age because of the smaller sample size (18, 19, 24, 25). The acute symptom spectrum and therapeutic approach were similar in all studies; however, variations were observed in the LOS. In our cohort, the median LOS was 12 days (IQR, 10–13 days), which was considered a prolonged hospitalization (>7 days) in other studies (17). In similar studies, the LOS varied between 5 and 11 days (19, 24, 26–29). With the evolving COVID-19 pandemic, the clinical phenotype of MIS-c and the severity of the condition are the major factors affecting the LOS (16, 17, 24).

According to our data, at the 2-week follow-up, almost half of the children (*N* = 10, 47.6%) experienced exercise intolerance with secondary-induced tiredness after any physical activity. Additionally, three children (14.3%) could not walk for more than 15 min or climb the stairs, and four (19%) had physical activity unrelated persistent fatigue. Within 2 months, improvement in exercise capacity was observed with a 5-fold and 2-fold reduction in physical intolerance (*N* = 2, 9.5%) and physical activity-induced fatigue (*N* = 5, 23.8%), respectively. However, no children reported physical activity-unrelated fatigue at the 2-month follow-up. Similarly, post-inflammatory physical exercise intolerance was observed in other studies. Kahn et al. reported post-acute phase fatigue in 22% of the study population, with reduced exercise capacity in 7% of children at the 2-week follow-up. A significant reduction (14%) in fatigue was observed at the 8-week follow-up, whereas the reduced exercise capacity remained constant (18). Penner et al. also reported a significant reduction in functional exercise capacity, with 65% of the children scoring below the 3rd percentile in the 6-minute walking test 6 weeks after MIS-c. Alarmingly, continuous poor performance on the 6-minute walking test was observed in 45% of patients even 6 months post hyperinflammatory condition (19). Notably, a more positive outcome was recently reported by Ziebell et al., who analyzed cardiopulmonary exercise data with peak oxygen consumption (peak VO_2_) detection in children with MIS-c and viral/idiopathic myocarditis 3–6 months after an acute event. No statistically significant differences in exercise capacity were observed between the groups, indicating a possible return to the previous level of daily functioning after MIS-c (30). Similarly, a recent study by Chakraborty et al., showed normal exercise capacity in all patients who underwent a graded-exercise stress test (GXT) 4–6 months after acute MIS-c (31).

Various factors may cause physical exercise intolerance and fatigue in children after SARS-CoV-2-induced hyperinflammation. However, the severity of acute MIS-c (cardiac involvement, hypotension, shock, and necessity for admission to the PICU with inotropic support or mechanical ventilation) plays an important role in the long-term consequences. Recently, the term “*Critical illness-associated weakness (CI-AW)*” has been frequently used to describe a group of neuromuscular disorders, including critical illness polyneuropathy (CIP) and critical illness myopathy (CIM), as complications of severe disease. Common risk factors for *CI-AW* development include hyperinflammation of different etiologies, multi-organ failure, prolonged mobility restrictions, hyperglycemia, and long-term use of glucocorticoids and/or neuromuscular blocking agents (32). Moreover, recent reports on acute glucocorticoid-induced proximal limb muscle myopathy in children and adults have emerged, raising awareness about the potential role of MIS-c baseline treatment in persistent symptom development (33, 34). Additionally, circumstances related to pandemic restrictions (lack of physical activity and a sedentary lifestyle) should be considered when reduced exercise capacity is observed.

Evaluation of cardiovascular events over time showed significant improvement in our cohort, with complete recovery (normal levels of cardiac markers, no residual changes in ECG and/or echo) within 2 months after MIS-c. Interestingly, similar trends were observed in other studies. Farooqi et al. conducted a similar study by following 45 children over 9 months. In the acute phase, 80% and 44% of the children had mild and moderate to severe echocardiographic abnormalities, respectively, including coronary abnormalities, with great response to treatment and rapid resolution of cardiac findings at first follow-up (1–4 weeks) in more than 72% of children (25). Additionally, a study from the United States of America showed significant improvement in cardiac function with an almost 16-fold reduction in ventricular systolic dysfunction and a 4-fold reduction in coronary artery aneurysms within 2 weeks after MIS-c (35). The extensive debate regarding the best possible scenario for the gradual resumption of physical activity after MIS-c has emerged, given the severe cardiac damage. Currently, the American Academy of Pediatrics (AAP) recommends exercise restrictions for a minimum of 3–6 months after MIS-c, with the cardiologist's conclusion for resuming any physical activities (36). Cardiac MRI (CMR) has recently become a helpful diagnostic tool for detecting and evaluating post-inflammatory myocardial injury (37). Generally, the CMR results were encouraging, with findings mostly depending on the examination time. For example, diffuse myocardial edema without evidence of late gadolinium enhancement was observed on CMR performed during the acute phase of MIS-c, excluding typical signs of viral myocarditis (38). Additionally, CMR performed 3 months after the resolution of MIS-c showed no permanent signs of myocarditis or fibrosis (39).

Considering the neurological sequelae, an interesting phenomenon was observed in both acute and post-acute phases. During the hospitalization, three children (14.3%) complained of abnormal sensations (burning and/or itching) on the scalp without objective evidence of cutaneous disease. One patient's (4.8%) complaints persisted throughout the follow-up with concurrent accompanying headaches. The patient consulted a dermatologist and neurologist; however, no pathology was found. To the best of our knowledge, there have been no reports of persistent MIS-c-related dysesthesia in children. In contrast, post-COVID-19 associated peripheral skin neuropathies have been reported in adults. Starace et al., reported that trichodynia (pain or burning sensation limited to the hair) was present in 58.4% of COVID-19 post-infectious patients presenting to specialized hair clinics with complaint resolution within 4–5 weeks (40). Interestingly, trichodynia in post-COVID-19 patients has been associated with persistent headaches, showing a similar pattern to that observed in our patients with dysesthesia.

Our study has several strengths. First, all patients with suspected or confirmed MIS-c were transferred to the CCUH because our hospital is the only tertiary-level pediatric medical institution in Latvia; this ensured all patients with MIS-c were identified and included in the study cohort. Second, this was a prospective longitudinal cohort study, implying that we could evaluate all patients in real time with consecutive follow-up visits according to the timeframe. Last, all visits were organized in person with a subsequent cardiologist's consultation and laboratory testing, ensuring objective and comprehensive patient evaluation.

This study had some limitations. First, no control group was included; therefore, this burdens the data interpretation in the context of other hyperinflammatory states or infectious diseases. Second, the small sample size limited the possibility of conducting in-depth statistical analyses and comparing the different patient groups. Thirdly, no validated tools were included to objectively detect a persistent symptom spectrum in children after MIS-c in this study. In addition, some diagnostic limitations also were seen. As described above, part of the children was transferred to CCUH from regional hospitals, meaning that initial laboratory tests in some cases were not available or were incomplete. Moreover, this also delayed initial cardiological investigation, potentially making the acute baseline data interpretation more difficult.

Despite these limitations, our study provides comprehensive insights into the clinical, laboratory, and cardiological course of MIS-c from the onset of the acute condition up to 6 months after hospitalization. Therefore, our data may be used in further studies to explore the nature and consequences of this postinfectious hyperinflammatory condition.

Currently the work on the follow-up program continues by dynamic patient monitoring. Moreover, validated multidimensional tools and control groups have been implemented to provide better insights into the long-term sequelae of MIS-c.

## Conclusions

This study found that most persistent symptoms were reported within the first 2 weeks after the acute phase, with decreased physical activity tolerance and activity-provoking fatigue as the main features. Furthermore, a positive trend was observed at each follow-up visit as the spectrum of the children's complaints decreased, enabling them to return to their daily activities. We believe that our study makes a significant contribution to the literature because it is the first report of persistent MIS-c-related dysesthesia, which clinicians can adopt in actual clinical settings to improve the quality of life of the affected patients.

## Data Availability

The raw data supporting the conclusions of this article will be made available by the authors, without undue reservation.
